# ﻿A new species of *Parasesarma* (Brachyura, Sesarmidae) from Western Australia, with a key to the species from Australia

**DOI:** 10.3897/zookeys.1255.162897

**Published:** 2025-10-13

**Authors:** Adnan Shahdadi, Andrew M. Hosie, Ana Hara, Benny K. K. Chan

**Affiliations:** 1 Biodiversity Research Center, Academia Sinica, Taipei 115, Taiwan Biodiversity Research Center, Academia Sinica Taipei Taiwan; 2 Collections and Research, Western Australian Museum, Welshpool, Western Australia 6106, Australia Collections and Research, Western Australian Museum Welshpool Australia

**Keywords:** Crustacea, COX1, mangrove crab, molecular taxonomy, morphology, *Parasesarma otiense*

## Abstract

Nine species of *Parasesarma* are currently recorded from continental Australian mangroves. The present study describes a new species, *P. otiense***sp. nov.**, from Western Australia. *Parasesarma otiense***sp. nov.** occurs sympatrically with *P. hartogi* and *P. holthuisi* in the mangroves of Exmouth Gulf. In both COX1 and 16SBI trees, the new species is well nested within *Parasesarma*, but divergent from other species. Morphologically, the new species is distinct from other *Parasesarma* in having eight asymmetric tubercles with the distal slope longer than the proximal slope on the upper surface of the cheliped dactylus. *Parasesarma otiense* is the tenth species of *Parasesarma* recorded from continental Australia, taking the number of species assigned to the genus *Parasesarma* to 59. A key to the species of *Parasesarma* known from Australian waters is provided to aid in their identification.

## ﻿Introduction

Brachyuran crabs of the genus *Parasesarma* De Man, 1895 are among the most common components of mangroves and estuaries (Lee 1998, 2015) and this genus has been the subject of significant recent taxonomic research. Based on molecular and morphological analyses, Shahdadi and Schubart (2018) transferred most species of *Perisesarma* De Man, 1895 to *Parasesarma*, and Shahdadi et al. (2020) transferred most of the long-legged species of *Parasesarma* to a new genus *Leptarma* Shahdadi, Fratini & Schubart, 2020. Currently, *Parasesarma* includes 58 species distributed across the Indo-West Pacific, with highest species diversity in Southeast Asia (Shahdadi et al. 2018, 2020, 2023).

Nine species of *Parasesarma* have been recorded from continental Australian mangrove habitats, with approximately half of the species endemic to either the eastern or western coasts, with some potential overlap zones along the northern coast between the Kimberley region of Western Australia to the Gulf of Carpentaria and eastern Cape York Peninsula in Queensland (Davie 1985). *Parasesarma lividum* (A. Milne-Edwards, 1869), *P. brevicristatum* (Campbell, 1967), and *P. erythodactyla* (Hess, 1865) have been recorded from the east coast (Campbell 1967; Davie 1993; Shahdadi et al. 2019). *Parasesarma longicristatum* (Campbell, 1967) and *P. messa* (Campbell, 1967) have a wider distribution from the east to the north coasts (Campbell 1967; Shahdadi et al. 2018). *Parasesarma darwinense* (Campbell, 1967), *P. austrawati* Shahdadi, Davie & Schubart, 2019, *P. holthuisi* (Davie, 2010), and *P. hartogi* Davie & Pabriks, 2010 have been recorded from northern to western coasts (Campbell 1967; Davie 2010; Davie and Pabriks 2010; Shahdadi et al. 2019). In addition to these species, *P. sigillatum* (Tweedie, 1950) is endemic to the Australian Indian Ocean Territory of the Cocos (Keeling) Islands (Ng et al. 2016).

The integrated molecular and morphological analyses of recent studies (e.g. Shahdadi et al. 2018, 2020; Shih et al. 2023) have provided a framework for new taxonomic research by clarifying the placement and the morphological delimitation of many species that were described during the 19^th^ and early 20^th^ centuries. The present study uses sequence data from specimens across a range of species and integrates this with morphology to describe a previously unknown species.

## ﻿Material and methods

Specimens were collected during different expeditions conducted by the Western Australian Museum: NCB Exmouth Muirons in 2016, Bush Blitz Cape Range in 2019, Environs Kimberley Broome in 2023, and Kimberley Reef Connect in 2023. Specimens were collected by hand from different localities (see material examined and Suppl. material 1) along the intertidal fringing mangroves and associated mud flats during low tides. The specimens were fixed in 96% ethanol and preserved in 75% ethanol. They were transferred to the Western Australian Museum (**WAM**), Perth, for further morphological examination and tissue subsampling for DNA extraction. All specimens are housed in the WAM or the Biodiversity Research Museum, Biodiversity Research Center, Academia Sinica, Taiwan (**ASIZ**).

Abbreviations used are as follows: **bp**: base-pairs; **coll.**: collected; **cl**: carapace length along the midline; **cw**: maximum carapace width; **P2–P5**: pereiopods 2–5, respectively (first to fourth ambulatory legs, respectively); **G1**: male first gonopod. Measurements are in millimetres (mm). The description is based on the holotype male with ranges and variations given in parentheses for paratypes, followed by female specific characters observed in the female paratype.

**Figure 1.**
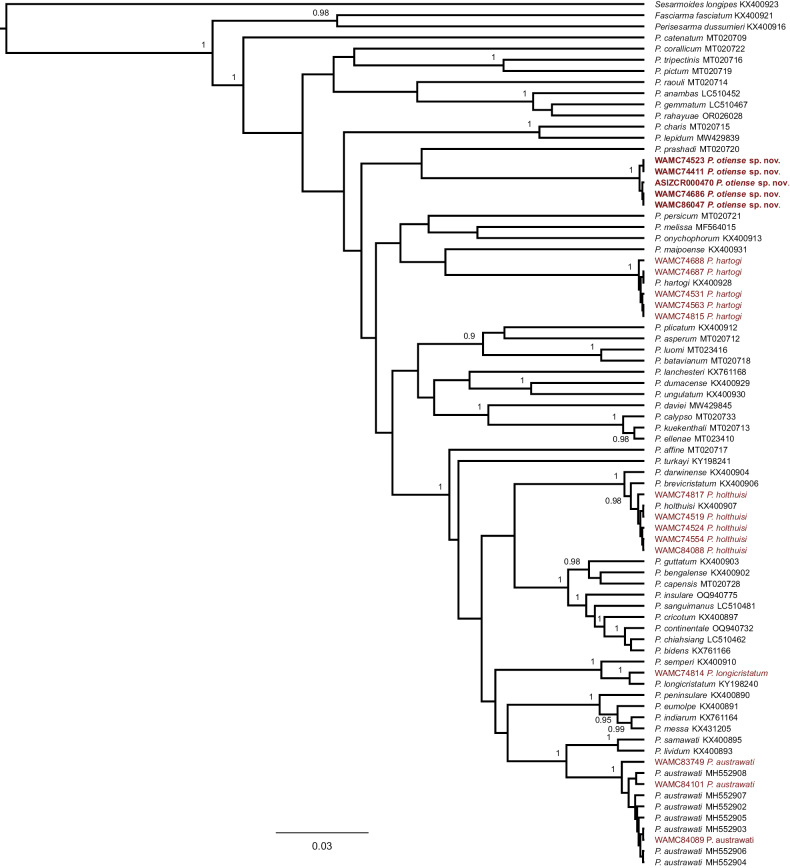
Bayesian Inference phylogram constructed in BEAST 2.7.7 for COX1 sequences of *Parasesarma*. The numbers behind the nodes refer to the support values (posterior probability) (posterior probabilities under 0.90 are not shown). Sequences belonging to *Sesarmoides longipes*, *Fasciarma fasciatum*, and *Perisesarma dussumieri* were used as outgroups. The numbers in front of species names are GenBank accession numbers. Sequences obtained in the present study are shown in red and the newly described species are in bold font (the numbers left to species names are Western Australian Museum numbers).

**Figure 2.**
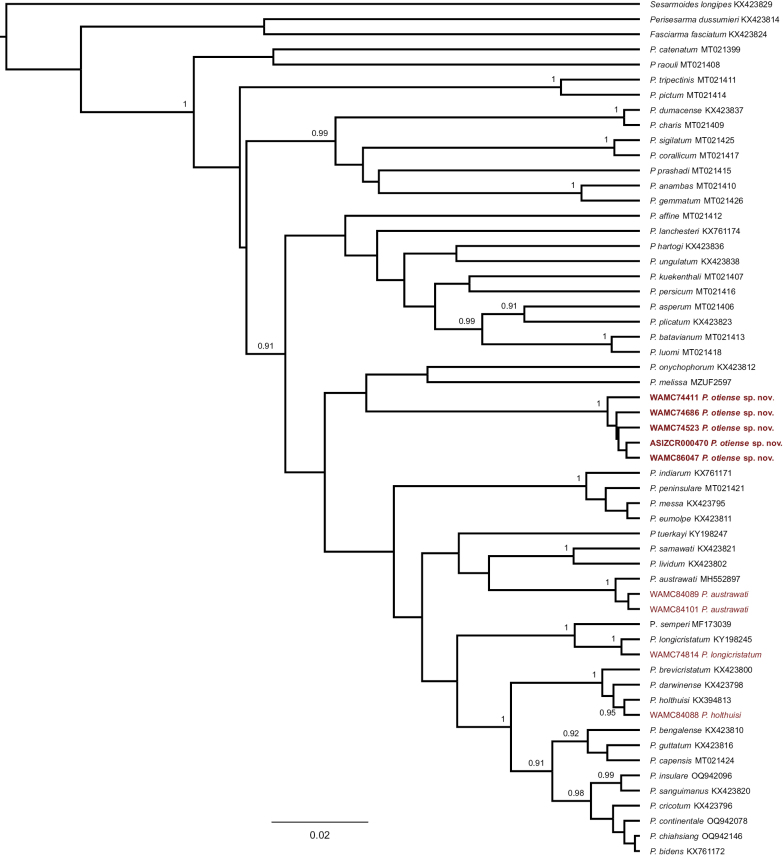
Bayesian Inference phylogram constructed in BEAST 2.7.7 for 16S sequences of *Parasesarma*. The numbers behind the nodes refer to the support values (posterior probability) (posterior probabilities under 0.90 are not shown). Sequences belonging to *Sesarmoides longipes*, *Fasciarma fasciatum*, and *Perisesarma dussumieri* were used as outgroups. The numbers in front of species names are GenBank accession numbers. Sequences obtained in the present study are shown in red and the newly described species are in bold font (the numbers left to species names are Western Australian Museum numbers).

Genomic DNA was isolated from muscle tissue using the Qiagen DNeasy extraction kit (Qiagen, Hilden, Germany) following the manufacturer’s protocol. A partial segment of the mitochondrial protein-coding gene cytochrome c oxidase subunit 1 (**COX1**), corresponding to the barcode region (Hebert et al. 2003), and a partial segment of 16S ribosomal DNA (16S) were selected as the most commonly used genetic markers in species delimitation in *Parasesarma* (e.g. Shahdadi et al. 2018). The polymerase chain reactions (**PCRs**) were performed using dgLCO1490 5′-GGTCAACAAATCATAAAGAYATYGG-3′ as forward primer and dgHCO2198 5′-TAAACTTCAGGGTGACCAAARAAYCA-3′ as reverse primer (Meyer 2003) for COX1; and 16L29 5′-YGCCTGTTTATCAAAAACAT-3′ as forward primer and 6H11 5′-6H11 AGATAGAAACCRACCTGG-3′ as reverse primer (Schubart 2009) for 16S. The PCR reactions were performed using 12.5 μl ThermoScientific DreamTaq Green PCR Master Mix (2×), 0.75 μl of each primer (10 μM), 1 μl of template DNA, and 10 μl of distilled water. The PCRs were conducted in a DNA Thermal Cycler T100 (Bio-Rad, Richmond, CA, USA) with the following profiles: an initial 7 cycles of 25 s at 95 °C, 25 s at 52 °C and 40 s at 72 °C; followed by 35 cycles of 25 s at 95 °C and annealing for 25 s at 46 °C for COI and 48 °C for 16S, 40 s at 70 °C for extension; and a final extension step of 7 min at 72 °C. New sequences were submitted to GenBank (https://www.ncbi.nlm.nih.gov/) and are available under accession numbers (Table 1). Sequences were assembled, proofread, and the primer regions were removed using Geneious Prime (https://www.geneious.com). Further quality control was undertaken through checking for stop codons in the translated sequence and checking for matches with non-target taxa on Blast (https://blast.ncbi.nlm.nih.gov/Blast.cgi). Sequences of other *Parasesarma* were downloaded from GenBank (https://www.ncbi.nlm.nih.gov/genbank/) and used for phylogenetic analyses. *Sesarmoides longipes* (Krauss, 1843), *Fasciarma fasciatum* (Lanchester, 1900), and *Perisesarma dussumieri* (H. Milne Edwards, 1853) were used as outgroups (see phylogenetic trees in Shahdadi et al. 2018, 2020) (for the accession numbers see Figs 1, 2, the phylogenetic trees). The sequences were aligned with ClustalW (Thompson et al. 1994) implemented in BioEdit 7.0.5 (Hall 1999).

**Table 1.** GenBank accession numbers for the sequences generated for this study.

Specimen	COI	16S
*Parasesarma austrawati*		
WAM C83749	PV871721	
WAM C84089	PV871717	PV882444
WAM C84101	PV871718	PV882443
*Parasesarma hartogi*		
WAMC74531	PV871707	
WAMC74563	PV871709	
WAMC74687	PV871711	
WAMC74688	PV871712	
WAMC74815	PV871714	
*Parasesarma holthuisi*		
WAMC74519	PV871704	
WAMC74524	PV871706	
WAMC74554	PV871708	
WAMC74817	PV871715	
WAMC84088	PV871716	PV882442
*Parasesarma longicristatum*		
WAMC74814	PV871713	PV882441
*Parasesarma otiense* sp. nov.		
WAMC74411	PV871703	PV882436
WAMC74523	PV871705	PV882440
WAMC74686	PV871710	PV882437
WAMC86047	PV871719	PV882438
ASIZCR000470	PV871720	PV882439

Available sequences of COX1 cover more species of *Parasesarma*, compared to the available sequences of 16S. We, therefore, conducted phylogenetic analysis for each gene separately. To address the phylogenetic positions of the new material, a Bayesian Inference (BI) was conducted in BEAST 2.7.7 (Drummond and Rambaut 2007) for each gene. We used the Yule Model (as prior for tree model) and a strict clock model. Markov Chains were run for 10 million generations, sampling every 1,000 iterations and discarding the first 10% as burn-in. The remaining 9,000 trees were used to calculate the maximum clade credibility tree in Tree Annotator (part of the BEAST package). The best evolutionary models were TIM2+F+I+G4 for COX1, and TIM+F+I+G4 for 16S as determined by using ModelFinder (Kalyaanamoorthy et al. 2017) through the IQ-TREE web server (http://iqtree.cibiv.univie.ac.at/?user=guest&jobid=241119083607) (Trifinopoulos et al. 2016) based on BIC scores. To calculate genetic distances (*p*-distance) we used MEGA X (Kumar et al. 2018).

## ﻿Results

### ﻿Phylogeny

In the present study we obtained 19 sequences of COX1 (three for *P. austrawati*, one for *P. longicristatum*, five for *P. hartogi*, five for *P. holthuisi*, and five for the new species) and nine sequences of 16S (two for *P. austrawati*, one for *P. holthuisi*, one for *P. longicristatum*, and five for the new species). Phylogenetic analyses confirmed the morphological identifications of *P. austrawati*, *P. hartogi*, *P. holthuisi*, and *P. longicristatum* as the new sequences of these species grouped stably with the sequences of their types (Figs 1, 2). In both COX1 and 16S trees, however, some sequences formed a well-supported sub-clade that is genetically divergent from other species of *Parasesarma*. This sub-clade is therefore identified as a new species. Although well nested within the clade of *Parasesarma* with a well-supported basal node, the COX1 and 16S phylogenies were not able to find any close phylogenetic allies of this new species.

*Parasesarma prashadi* (Chopra & Das, 1937) was sister to *P. otiense* sp. nov. in the COX1 tree, with a *p*-distance of 8.4%. In the 16S tree, *P. onychophorum* (De Man, 1895) and *P. melissa* (De Man, 1887) formed a clade as sister to the new species. *Parasesarma otiense* sp. nov. was 7% divergent from the *P. onychophorum* + *P. melissa* clade, while *P. onychophorum* and *P. melissa* were 4.2% divergent from each other.

### ﻿Systematic account

**Family Sesarmidae Dana, 1851**

**Genus *Parasesarma* De Man, 1895**

#### ﻿ Parasesarma otiense sp. nov.

Taxon classificationAnimaliaDecapodaSesarmidae

2BBCB7FE-AA51-5AD3-B1B6-DEFC51035879

https://zoobank.org/515F271B-6A51-4A70-A087-382922061A5D

Figs 2, 3, 4

##### Material examined.

***Holotype*.** Australia • WAM C74523, male (14.0 × 11.0); Western Australia, Exmouth Gulf, Bay of Rest mangroves (22°18'54"S, 114°7'33"E); 21 June 2019; Bush Blitz Cape Range; coll. Hosie, A. M. & Hara, A. ***Paratypes*.** • WAM C74411, male (12.1 × 9.8); Western Australia, Exmouth Gulf, Bay of Rest mangroves (22°18'44"S, 114°7'38"E); 4 June 2016; NCB Exmouth Muirons; coll. Hosie, A. M. & Hara, A. • WAM C74686 male (6.5 × 5.4); Western Australia, Exmouth Gulf, Bay of Rest mangroves (22°18'54"S, 114°7'33"E); • WAM C86047, Female (9.4 × 7.6); Western Australia, Exmouth Gulf, Bay of Rest mangroves (22°18'44"S, 114°7'38"E); 4 June 2016; NCB Exmouth Muirons; coll. Hosie, A. M. & Hara, A. • ASIZCR000470 male (8.6 × 6.8), Western Australia, Exmouth Gulf, Bay of Rest mangroves (22°18'44"S, 114°7'38"E); 4 June 2016; NCB Exmouth Muirons; coll. Hosie, A. M. & Hara, A.

##### Diagnosis.

Ambulatory legs relatively long, P4 longest, ~1.7 × cw. Carapace rectangular, broader than long, dorsal surface smooth, front moderately deflexed, shallowly sinuous in dorsal view, median postfrontal lobes as wide as lateral ones. Eyestalk longer than wide, cornea wider than eyestalk. Chelipeds without subdistal spine on dorsal border of merus; male chela with 2 transverse pectinated crests on the upper surface of palm, dactylus with 8 asymmetric tubercles with proximal slope shorter than distal slope, tubercles with transverse keel and wrinkles. Male pleon triangular, somite 2 medially longer than lateral edges. G1 stout, straight, apical corneous process relatively long, bent at an angle of ~45° to vertical axis, aperture subterminal.

##### Description

**Figure 3.**
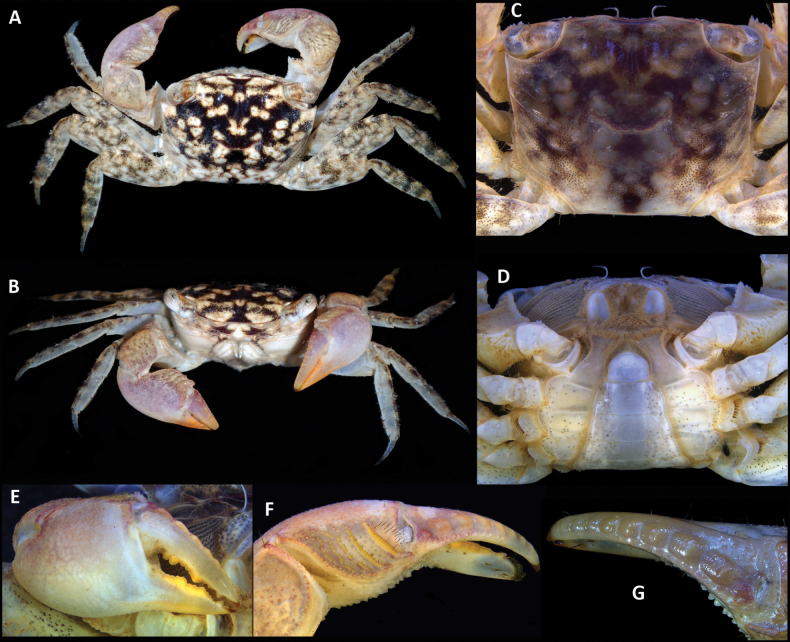
*Parasesarma otiense* sp. nov., holotype, WAM C74523, male (14.0 × 11.0), Western Australia, Exmouth Gulf, Bay of Rest mangrove. A. Dorsal habitus; B. Frontal view; C. Carapace, dorsal view; D. Pleon and mouth; E. Right chela, outer view; F. Left chela, dorsal view; G. Dactylus of right chela, dorsal view.

**Figure 4.**
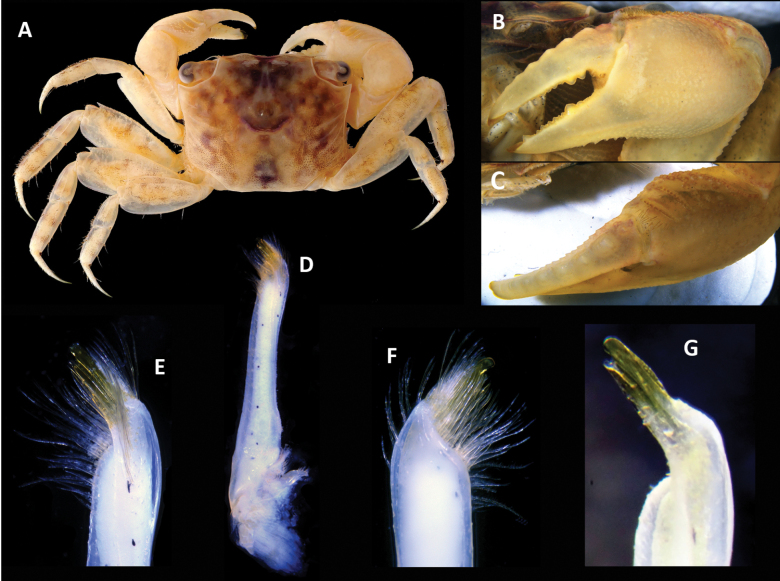
*Parasesarma otiense* sp. nov., paratype, WAM C74411, male (12.1 × 9.8), Western Australia, Exmouth Gulf, Bay of Rest mangrove. A. Dorsal habitus; B. Left chela, outer view; C. Left chela, dorsal view; D–G. Left G1; D. Full dorsal view; E. Distal tip, dorsal view; F. Distal tip, ventral view; G. Distal tip, dorsal view, denuded.

**Figure 5.**
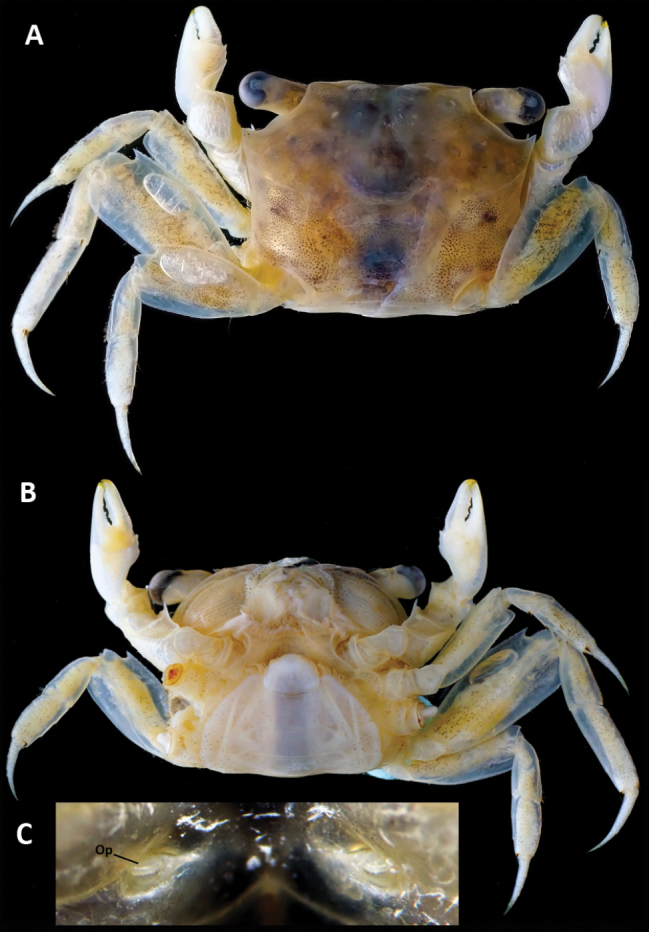
*Parasesarma otiense* sp. nov., paratype, WAM C86047, Female (9.4 × 7.6), Western Australia, Exmouth Gulf, Bay of Rest mangrove. A. Dorsal habitus; B. Ventral habitus; C. Vulvae (Op = operculum).

**(morphometrics based on the holotype but with variation and ranges in parentheses).** Carapace (Figs 3A, C, 4A, 5A) rectangular, broader than long, greatest width between exorbital angles, cw/cl = 1.27 (1.20–1.27); dorsal surface smooth and shiny (Fig. 3A, C); front in holotype ~0.55 × cw (0.55–0.58), moderately deflexed, shallowly sinuous in dorsal view; postfrontal lobes distinct, median lobes as broad as lateral lobes, separated by well-pronounced furrow (Fig. 3A, C); dorsal regions well indicated, gastric region demarcated, cardiac region not well separated from intestinal region; lateral branchial ridges prominent; anterolateral margin with sharp exorbital angle directed anteriorly; lateral margins straight with no indication of epibranchial tooth, edged with row of short setae. Eyestalk longer than wide, cornea wider than eyestalk (Fig. 3C).

Chelipeds similar (Figs 3A, B, 4A); chelae (Figs 3E, F, G, 4B, C) large, palm length 0.76 × cw, robust, palm length 1.77 × palm width. Merus with finely granulate dorsal border, but no subdistal spine; ventral border granulate; anterior border granulate, with large subdistal spine; inner face smooth with a longitudinal row of setae. Upper surface of palm with 2 transverse pectinated crests and 2 or 3 crests consisting of granules (Figs 3F, 4C), distal (primary) crest composed of 14 or 15 tall teeth (varying on opposite claws of holotype), secondary crest well developed, with 13 teeth; both crests ending on inner side in short swollen, tubercular ridge and several small granules at outer side; upper margin of palm distal to pectinated crests with some setae (Fig. 3F); outer surface of palm with fine granules, with granules forming a line on fixed finger (Figs 3E, 4B); inner surface of palm with granules but no vertical ridge; ventral border of chela sinuous with granules; length of cutting margin of fixed finger of holotype ~0.4 × length of entire propodus. Dactylus (Figs 3E, F, G, 4B, C) straight in outer view but slightly curved inward, ~0.6 × propodus length in holotype; dorsal surface bearing 8 rounded asymmetric tubercles with proximal slope shorter than distal slope, distinct to tip, tubercles with transverse keel and wrinkles, creating step-like shape for each tubercle, proximal tubercles positioned at inner part of upper dactylar face, row of small rounded tubercles on proximal half of inner edge of dorsal surface; fingers with chitinous tips, cutting edge of both fingers with a series of variably sized teeth.

Ambulatory legs (Figs 3A, 4A, 5A) relatively long; P4 longest, length (ischium–dactylus) 1.66 × cw (1.62–1.78), merus with anterior margin crenulated, ~2.3 × as long as wide, propodus ~3.2 × as long as wide, dactylus length ~0.8 × length of propodus. Male pleon (Fig. 3D) triangular; telson slightly shorter than basal width, slightly longer than somite 6; somite 6 longer than others; somite 5 and 4 trapezoidal, somite 3 widest, laterally convex, somite 2 medially longer than lateral edges. G1 (Fig. 3D–G) stout, straight, stem triangular with blunt angles in cross-section; apical corneous process relatively long, bent at an angle of ~45° to vertical axis, tip rounded, aperture subterminal.

Females (Fig. 5) with proportionally smaller chelipeds than males, palm length 0.50 × cw; pectinated crest absent on palm, replaced by rows of granules; dactylus with 8 small but distinct, round tubercles. Pleon (Fig. 5B) broad, rounded, broadest at somites 3 and 4, fringed with long setae, touching coxae of ambulatory legs. Vulva (Fig. 5C) in depression on anterior edge of sternite 6, operculum oval, parallel and touching line of sternite 5, oval operculum in anterior part of vulva.

##### Etymology.

The species name is derived from the Latin noun *otium*, meaning rest, and the gender-neutral suffix, -*ense*, in reference to the type locality, the Bay of Rest in Exmouth Gulf, Western Australia.

##### Habitat.

Intertidal fringing mangrove and associated mud flat.

## ﻿Discussion

*Parasesarma otiense* sp. nov. co-occurs with *P. hartogi* and *P. holthuisi* (see comparative material listed in Suppl. material 1) within the Bay of Rest but differs in carapace morphology, with no sign of epibranchial teeth (Fig. 3C). In contrast, *P. hartogi* (see Davie and Pabriks 2010: fig. 1B) has a distinct epibranchial projection and *P. holthuisi* (see Davie 2010: fig. 1B) has a small but clearly incised epibranchial tooth. The cheliped dactylus in *P. otiense* sp. nov. is straight with eight distinct rounded, asymmetric tubercles (Fig. 3E–G), rather than downcurved with low tubercles in *P. hartogi* (see Davie and Pabriks 2010: fig. 2A) or with 12 or 13 transversely broadened tubercles (Davie 2010: fig. 2B) *in P. holthuisi*.

Of the eight other species found in Australian territories, *P. austrawati*, *P. brevicristatum*, *P. darwinense*, *P. lividum*, *P. longicristatum*, and *P. messa* all bear a well-developed epibranchial tooth on each side of the carapace (Campbell 1967; Shahdadi et al. 2018, 2019). While *P. erythodactyla* from eastern Australia (Davie 1993) and *P. sigillatum* from the Cocos (Keeling) Islands (Ng et al. 2016) lack epibranchial teeth but can be distinguished from *P. otiense* sp. nov. by the number and morphology of the tubercles on the cheliped dactylus. *Parasesarma erythodactyla* has 23–26 symmetrical tubercles (see Davie 1993: fig. 3A, C), and *P. sigillatum* has 16–18 triangular tubercles (see Ng et al. 2016: fig. 3E–H), compared with only eight asymmetric tubercles in *P. otiense* sp. nov. (Fig. 3E–G).

*Parasesarma ellenae* (Pretzmann, 1968) is distributed in the South Pacific islands and has six or seven rounded asymmetric tubercles on cheliped dactylus (Shahdadi et al. 2021). *Parasesarma kuekenthali* (De Man, 1902) has eight or nine asymmetric tubercles on the cheliped dactylus and occurs in Southeast Asia from the Philippines to the Indonesian islands of Sulawesi and Halmahera (Shahdadi et al. 2021). The tubercles in these two species are, however, different from the new species as the proximal slope is longer than the distal slope in *P. ellenae* (see Shahdadi et al. 2021: fig. 9E) and *P. kuekenthali* (see Shahdadi et al. 2021: fig. 14D), whereas in *P. otiense* sp. nov. the distal slope is longer (Fig. 3E).

Understanding the diversity of Australian *Parasesarma* has been hindered by a complicated taxonomic history of the group (Shahdadi et al. 2018). Molecular phylogenetics has supported the distinction of many morphologically similar or cryptic species and has clarified some characters as well as distributions. In the case of *P. otiense* sp. nov., the phylogenetic results (both COX1 and 16S) support the morphological differences observed (Figs 1, 2). The genetic distances between the new species and the closest species in both trees are much higher than the distances between previously known species pairs of *Parasesarma* (Shih et al. 2023) as well as many other crustacean sister species (Matzen da Silva et al. 2011; Chu et al. 2015).

While not central to this study, the identification of *P. longicristatum* from Exmouth Gulf represents a considerable extension of the known range south from Admiral Island in the Kimberley region. Previously, the known range of *P. longicristatum* extended from Moreton Bay, Queensland, across northern Australia, westwards into the Kimberley region of Western Australia, and this new record makes this species the most widespread member of the genus in Australia (Shahdadi et al. 2018).

Although identifying species within the genus is not a trivial endeavour, the key below provides clear and relatively simple steps to aid in the identification of the known Australian *Parasesarma*.

### ﻿Key to the *Parasesarma* known from Australian territories

**Table:** 

1	Carapace without trace of epibranchial teeth	**2**
–	Carapace with epibranchial tooth or angle	**4**
2	Cheliped dactylus with 23–26 symmetrical, transversely broadened tubercles	***P. erythodactyla***
–	Cheliped dactylus with < 20 tubercles	**3**
3	Cheliped dactylus with 8 asymmetric tubercles	***P. otiense* sp. nov.**
–	Cheliped dactylus with 16–18, triangular tubercles	***P. sigillatum***
4	Carapace epibranchial tooth not fully developed, forming prominent angle	***P. hartogi***
–	Carapace epibranchial tooth developed, clearly incised	**5**
5	Cheliped dactylus with up to 9 tubercles	**6**
–	Cheliped dactylus with 10 or more tubercles	**7**
6	Cheliped dactylus with 7 or 8 symmetrical, rounded tubercles; distal pectinate crest with up to 17 tall and broad teeth	***P. austrawati***
–	Cheliped dactylus with 8 or 9 asymmetrical tubercles; distal pectinate crest with ~25 teeth	***P. longicristatum***
7	Cheliped fixed finger short, length of cutting-edge ca 0.37 × length of propodus; dactylus with 10–13 irregularly shaped tubercles	***P* . *lividum***
–	Cheliped fixed finger longer, length of cutting-edge ca 0.40 × length of propodus; dactylar tubercles not forming irregular shapes in dorsal view	**8**
8	Cheliped dactylus with 10 or 11 symmetrical, subcircular tubercles	***P. brevicristatum***
–	Cheliped dactylus with 12 or more tubercles	**9**
9	Cheliped dactylus with 14–16 tubercles, but only proximal half of tubercles are prominent, distal tubercles barely discernible	***P. messa***
–	Cheliped dactylus tubercles generally of similar prominence throughout length	**10**
10	Cheliped dactylus with 12 or 13 broadened tubercles	***P. holthuisi***
–	Cheliped dactylus with 15 or 16 tubercles	***P. darwinense***

## Supplementary Material

XML Treatment for Parasesarma otiense

## ﻿Additional information

### Use of AI

No use of AI was reported.

### Author ORCIDs

Adnan Shahdadi https://orcid.org/0000-0001-5131-7700

Andrew M. Hosie https://orcid.org/0000-0002-5683-662X

Ana Hara https://orcid.org/0009-0001-9881-7937

Benny K. K. Chan https://orcid.org/0000-0001-9479-024X

### Data availability

All of the data that support the findings of this study are available in the main text or Supplementary Information.

## Supplementary materials

Supplementary material 1 Comparative material examined and sequenced Data type docx Explanation note Locality data for the comparative material examined during this study. https://binary.pensoft.net/file/1437056This dataset is made available under the Open Database License (http://opendatacommons.org/licenses/odbl/1.0/). The Open Database License (ODbL) is a license agreement intended to allow users to freely share, modify, and use this Dataset while maintaining this same freedom for others, provided that the original source and author(s) are credited.Adnan Shahdadi, Andrew M. Hosie, Ana Hara, Benny K. K. Chan

Supplementary material 2 Measurements Data type docx Explanation note Measurements of selected morphological features and relevant ratios used in the description of *Parasesarma otiense* sp. nov. https://binary.pensoft.net/file/1437057This dataset is made available under the Open Database License (http://opendatacommons.org/licenses/odbl/1.0/). The Open Database License (ODbL) is a license agreement intended to allow users to freely share, modify, and use this Dataset while maintaining this same freedom for others, provided that the original source and author(s) are credited.Adnan Shahdadi

Supplementary material 3 COX1BI tree in nexus format Data type nexus format file Explanation note Bayesian Inference tree of COX1 data. https://binary.pensoft.net/file/1437058This dataset is made available under the Open Database License (http://opendatacommons.org/licenses/odbl/1.0/). The Open Database License (ODbL) is a license agreement intended to allow users to freely share, modify, and use this Dataset while maintaining this same freedom for others, provided that the original source and author(s) are credited.Adnan Shahdadi

Supplementary material 4 16SBI tree in nexus format Data type nexus format file Explanation note Bayesian Inference tree of 16S sequence data. https://binary.pensoft.net/file/1437059This dataset is made available under the Open Database License (http://opendatacommons.org/licenses/odbl/1.0/). The Open Database License (ODbL) is a license agreement intended to allow users to freely share, modify, and use this Dataset while maintaining this same freedom for others, provided that the original source and author(s) are credited.Adnan Shahdadi
