# Supplementary material for: ﻿A new species of Parasesarma (Brachyura, Sesarmidae) from Western Australia, with a key to the species from Australia
Source: Zookeys. 2025 Oct 13;1255:275–90. doi: 10.3897/zookeys.1255.162897 (PMC12538216; doi:10.3897/zookeys.1255.162897)
Supplement: Supplementary material 1 — Comparative material examined and sequenced [file zookeys-1255-275_article-162897__-s001.docx]

**Supplementary File 1. Locality data of the specimens examined as comparative material.**

*Parasesarma* *austrawati*

• WAM C83749 male (14.3×11.5); Western Australia, East Montalivet Island (14°17’09"S, 125°18’18"E) • WAM C84089 female (23.8×19.0); Western Australia, Barred Creek mangroves (17°39’46.0"S, 122°12’01.5"E) • WAM C84101 female (24.4×19.8); Western Australia, Barred Creek mangroves (17°39’46.0"S, 122°12’01.5"E) •

*Parasesarma hartogi*

• WAM C74531, female (13.4× 17.7); Western Australia, Exmouth Gulf, Bay of Rest mangroves (22°18’44"S, 114°7’28"E) • WAM C74563, male (16.2×21.0); Western Australia, mangroves south of Doole Island (22°32’5"S, 114°9’44"E) • WAM C74687 male (18.3×23.8); same data as for preceding • WAM C74688, male (16.4×21.4); same data as for preceding • WAM C74815, male (20.2×15.5); same data as for preceding.

*Parasesarma holthuisi*

• WAM C74519, female (16.5×20.1); Western Australia, Exmouth Gulf, Bay of Rest mangrove (22°18’54"S, 114°7’33"E) • WAM C74524 (12.6×10.3); same data as for preceding • WAM C74554 male (16.7×20.2); Western Australia, Mangroves south of Doole Island (22°32’5"S, 114°9’44"E) • WAM C74817 male (14.2×17.6); same data as for preceding • WAM C84088 male (10.1×8.3); Western Australia, Barred Creek, mangroves (17°39’46.0"S 122°12’01.5"E).

*Parasesarma longicristatum*

• WAM C74814 female (8.4×6.7); Western Australia, Mangroves south of Doole Island (22°32’5"S 114°9’44"E). Specimens were collected by Hosie, A. M. & Hara, A.
