# Supplementary material for: ﻿A new species of Parasesarma (Brachyura, Sesarmidae) from Western Australia, with a key to the species from Australia
Source: Zookeys. 2025 Oct 13;1255:275–90. doi: 10.3897/zookeys.1255.162897 (PMC12538216; doi:10.3897/zookeys.1255.162897)
Supplement: Supplementary material 2 — Measurements [file zookeys-1255-275_article-162897__-s002.docx]

**Supplementary File 2.** Measurements of selected morphological features and relevant ratios used in the description of *Parasesarma otiense* **sp. nov.** All measurements are in millimetres

|  | **Specimen** | | | | |
| --- | --- | --- | --- | --- | --- |
| **Measurement** | **WAM C74523** | **WAM C74411** | **WAM C74686** | **WAM C86047** | **ASIZCR000470** |
| Carapace width (CW) | 14.00 | 12.10 | 6.50 | 9.40 | 8.60 |
| Carapace length (CL) | 11.00 | 9.80 | 5.40 | 7.60 | 6.80 |
| CW/CL | 1.27 | 1.23 | 1.20 | 1.24 | 1.26 |
| Front | 7.72 | 6.70 | 3.80 | 5.40 | 4.80 |
| Front/CW | 0.55 | 0.55 | 0.58 | 0.57 | 0.56 |
| Palm length (PL) | 10.60 | 9.40 | 3.40 | 4.70 | 5.80 |
| PL/CW | 0.76 | 0.78 | 0.52 | 0.50 | 0.67 |
| Palm width (PW) | 6.00 | 5.30 | 1.60 | 2.10 | 2.80 |
| PL/PW | 1.77 | 1.77 | 2.13 | 2.24 | 2.07 |
| Fixed finger | 4.00 | 3.20 | 1.20 | 1.90 | 2.20 |
| Palm dactylus (larger one) length | 6.40 | 5.50 | 1.60 | 2.80 | 3.40 |
| Telson length | 2.10 | 1.85 | 1.10 | 1.50 | 1.40 |
| Telson width | 2.30 | 2.20 | 1.40 | 1.90 | 1.80 |
| 6 pleon segment length | 1.80 | 1.50 | 1.00 | 2.00 | 1.30 |
| 6 pleon segment width | 4.00 | 3.90 | 2.10 | 5.90 | 2.90 |
| Pereopod 4 (P4) merus length | 9.00 | 7.80 | 4.30 | 5.90 | 5.90 |
| P4 merus width | 3.90 | 3.60 | 1.90 | 2.80 | 2.50 |
| P4 carpus length | 4.80 | 3.70 | 2.40 | 2.80 | 2.60 |
| P4 propodus length | 5.20 | 4.40 | 2.70 | 3.50 | 3.20 |
| P4 propodus width | 1.60 | 1.40 | 0.80 | 1.20 | 1.10 |
| P4 dactylus length | 4.20 | 3.80 | 2.20 | 3.00 | 2.90 |
| P4 length (ischium-dactylus) | 23.20 | 19.70 | 11.60 | 15.20 | 14.60 |
| P4 length/CW | 1.66 | 1.63 | 1.78 | 1.62 | 1.70 |
